# Non-restorative Sleep Caused by Autonomic and Electroencephalography Parameter Dysfunction Leads to Subjective Fatigue at Wake Time in Shift Workers

**DOI:** 10.3389/fneur.2019.00066

**Published:** 2019-02-05

**Authors:** Sofya Gorlova, Tomohisa Ichiba, Hiroshi Nishimaru, Yusaku Takamura, Jumpei Matsumoto, Etsuro Hori, Yoshinao Nagashima, Tsuyoshi Tatsuse, Taketoshi Ono, Hisao Nishijo

**Affiliations:** ^1^System Emotional Science, Graduate School of Medicine and Pharmaceutical Sciences, University of Toyama, Toyama, Japan; ^2^Personal Health Care Lab, KAO Corp, Tokyo, Japan; ^3^Department of Epidemiology and Health Policy, Graduate School of Medicine and Pharmaceutical Sciences, University of Toyama, Toyama, Japan

**Keywords:** subjective fatigue, electroencephalography, slow-wave sleep, delta wave, heart rate variability, total power

## Abstract

Sleep is a physiological state that plays important role in the recovery of fatigue. However, the relationship between the physiological status of sleep and subjective fatigue remains unknown. In the present study, we hypothesized that the non-recovery of fatigue at wake time due to non-restorative sleep might be ascribed to changes in specific parameters of electroencephalography (EEG) and heart rate variability (HRV) in poor sleepers. Twenty healthy female shift-working nurses participated in the study. Subjective fatigue was assessed using the visual analog scale (VAS) at bedtime and wake time. During sleep on the night between 2 consecutive day shifts, the EEG powers at the frontal pole, HRV based on electrocardiograms, and distal-proximal gradient of skin temperature were recorded and analyzed. The results indicated that the subjects with high fatigue on the VAS at wake time exhibited (1) a decrease in deep non-rapid eye movement (NREM) (stageN3) sleep duration in the first sleep cycle; (2) a decrease in REM latency; (3) a decrease in ultra-slow and delta EEG powers, particularly from 30 to 65 min after sleep onset; (4) a decrease in the total power of HRV, particularly from 0 to 30 min after sleep onset; (5) an increase in the very low frequency component of HRV; and (6) a smaller increase in the distal-proximal gradient of skin temperature, than those of the subjects with low fatigue levels. The correlational and structural equation modeling analyses of these parameters suggested that an initial decrease in the total power of HRV from 0 to 30 min after sleep onset might inhibit the recovery from fatigue during sleep (i.e., increase the VAS score at wake time) via its effects on the ultra-slow and delta powers from 30 to 65 min after sleep onset, stageN3 duration in the first sleep cycle, REM latency, and distal-proximal gradient of skin temperature. These findings suggest an important role of these physiological factors in recovery from fatigue during sleep, and that interventions to modify these physiological factors might ameliorate fatigue at wake time.

## Introduction

Sleep is a physiological state that plays important role in the recovery of fatigue (1). Extensive studies have reported some changes in the electroencephalography (EEG) power and heart rate variability (HRV) in various diseases (e.g., chronic fatigue syndrome, multiple sclerosis, and cancers) associated with sleep disturbances and fatigue. In chronic fatigue syndrome, patients exhibit sleep disturbance (2, 3), and changes in ultra-slow and delta powers as well as changes in HRV during sleep (4–7). Multiple sclerosis patients reported both sleep disturbance and fatigue, in which the former significantly contributed to the latter (8, 9). In cancer patients and cancer survivors, sleep disorders and fatigue are prevalent, and cancer-related fatigue is correlated with various subjective sleep parameters such as sleep quality (10). Interventions for optimizing sleep quality could lower fatigue in such patients (11). However, the direct relationship between fatigue and the objectively measured quality and quantity of sleep remains unknown (1, 12).

The association between shift work and fatigue in workers has been examined extensively. The problems related to rotational-shift work affect sleep and work performance (13–15). Nurses with greater fatigue and poorer sleep quality more often made decisions that they later regretted (16). However, there are individual differences in the sensitivity to shift work among shift workers (17). Our recent study on nurses found that the subjective fatigue at wake time while they worked the day off to day shift and day shift to day shift was significantly higher in poor sleepers than in good sleepers (18). These results suggest that certain physiological factors during sleep might be related to individual differences (i.e., good vs. poor sleepers), which might in turn affect subjective fatigue at wake time.

In the present study, we hypothesized that fatigue at wake time might be ascribed to “non-restorative sleep,” which is linked to specific changes in EEG and HRV parameters in poor sleepers. To investigate this issue, we analyzed the relationship between subjective fatigue at wake time and the physiological parameters during sleep in shift nurses, in the same situation as in our previous study (18) (i.e., the nurses worked the same shift schedule and slept at home). For this purpose, the physiological data were recorded from the nurses at their home while they worked similar shift work schedules.

## Materials and Methods

### Subjects

Twenty healthy female full-time nurses (mean age 35.0 ± 2.0 years; mean ± SEM) working at the Toyama University Hospital participated in the study. The nurses were neither pregnant nor breastfeeding. They were instructed to refrain from drinks that contain caffeine or alcohol after dinner. Data from one subject who consumed alcohol on the night of recording were excluded from the analysis, leaving a total of 19 subjects for further data analysis (mean age 34.7 ± 2.1 years). The inclusion criteria for subject selection were as follows: (1) female nurses who worked in the university hospital in a specific shift work pattern (see next section). The exclusion criteria for subject selection were as follows: (1) subjects who routinely took medicines such as sleeping pills; (2) subjects who were receiving medical treatment; (3) subjects who could not attach probes on own body by themselves; (4) subjects who had allergies to medical tapes; (5) subjects whose sleep period time were < 270 min on the experimental day; (6) subjects who took drinks including caffeine or alcohol in and after the dinner on the experimental day. All subjects were treated in strict compliance with the Declaration of Helsinki and the U.S. code of Federal Regulations for the Protection of Human Subjects. The experiments were conducted with the understanding and informed written consent of each subject, and approved by the Clinical Research and Ethics Committee at the University of Toyama.

### Study Schedule

**Figure 1:**
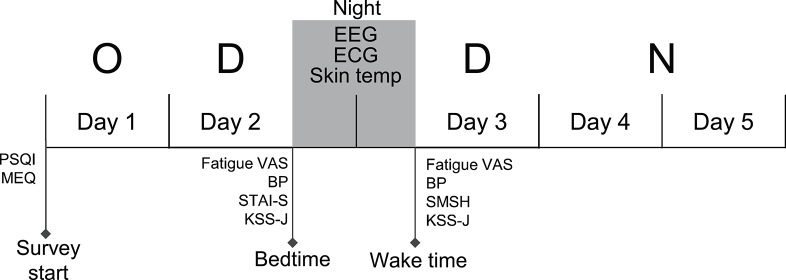
Experimental and shift schedule during the survey. The EEGs, ECGs, and skin temperature (Skin temp) were recorded on the night between consecutive dayshift (between day 2 and day 3). Fatigue and other subjective parameters were measured at bedtime on day 2 and at wake time on day 3, respectively. O, Day Off; D, Day shift; N, Night shift; PSQI, Pittsburgh Sleep Quality Index; MEQ, Morningness-Eveningness Questionnaire; STAI-S, state anxiety questionnaires of State-Trait Anxiety Inventory; VAS, visual analog scale; SMSH, Saint Mary's Hospital Sleep Questionnaire; KSS-J, Karolinska Sleepiness Scale (Japanese version); BP, blood pressure.

The data were recorded from nurses who were engaged in the following shift schedule; day off (day 1), 8 h day shift for 2 days (days 2 and 3), and one 18 h night shift (days 4 and 5) (the day off—day shift—day shift—night shift [ODDN] schedule) (Figure 1). Prior to the ODDN schedule, all subjects were trained to attach an EEG/electrocardiogram (ECG) device and skin temperature electrodes, and performed a trial night at home. We confirmed that all subjects could correctly record EEGs and ECGs in a trial recording. Furthermore, prior to the ODDN schedule, the subjects completed three self-report questionnaires including the Pittsburgh Sleep Quality Index (PSQI), and Morningness-eveningness questionnaire (MEQ). On the night of day 2, EEGs, ECGs, and skin temperature were recorded at home. On the physiological recording days (before bedtime on day 2 and after wake-up on day 3), the nurses rated their current anxiety using the state anxiety questionnaires in State-Trait Anxiety Inventory (STAI-S), subjective fatigue using the Visual Analog Scale (VAS), and subjective sleepiness using the Karolinska sleepiness scale (Japanese version) (KSS-J) before bedtime on day 2. Furthermore, after they woke on day 3, they also assessed their subjective sleep quality using the St. Mary's hospital (SMH) sleep questionnaire, and rated their subjective fatigue using the VAS and current subjective level of sleepiness using the KSS-J. The subjects were instructed to measure blood pressure on the physiological recording days (before bedtime on day 2 and after waking on day 3).

### Questionnaires

The PSQI is widely used for clinical purposes in order to assess subjective sleep quality over a 1 month period, and consists of 19 items in seven domains (19). The total score ranges from 0 to 21, with a lower score indicating better sleep quality. The MEQ consists of 19 items measuring the degree to which the respondent favors morning over evening (20), with high and low scores representing morningness and eveningness, respectively. The STAI-S is a commonly used inventory for measure of state anxiety levels (21).

Subjective fatigue was rated using the VAS (18, 22, 23). Subjective sleep quality (e.g., sleep depth, sleep quality, satisfaction) was rated using the SMH sleep questionnaire (24), and the current subjective level of sleepiness was rated using Karolinska sleepiness scale (Japanese version) (25).

### Physiological Recordings

The active/rest cycle and sleep/wake parameters were measured throughout the study period using the Actiwatch Spectrum Plus® (Philips Respironics, Bend Oregon). EEGs and ECGs were recorded during sleep at home using a single-channel portable EEG device (Brainwave Sensor ZA®; Proasist, Osaka, Japan) at a sampling rate of 128 Hz. The participants were instructed to place disposable surface electrodes at the center of the forehead and right mastoid process (for the EEG) and another two electrodes were placed on the chest to record ECGs (lead II).

The nocturnal skin temperature was recorded on the same night using nine temperature loggers (iButton® DS1920; Maxim Integrated, CA, USA). The temperature loggers were placed on the bilateral subclavian regions, center of the abdomen at 1 cm above the navel, bilateral lunate bones on the palmar side, center of the thigh on the bilateral limbs, and bilateral regions below the medial malleolus (26).

### Data Analysis

In accordance with the original criteria (27, 28), sleep stages were determined by visual inspection of the EEG data recorded from the frontal pole during each 30 s epoch by a clinical professional technologist, and classified into the four sleep stages; (1) awake (stageW), (2) rapid eye movement (REM) sleep (stageR), (3) light non-REM (NREM) sleep (stageN1 and stageN2), and (4) deep NREM sleep (stageN3). Simultaneous sleep recordings using the single-channel EEG device with single-channel EOGs (Brainwave Sensor ZA®) and conventional polysomnography according to the AASM Manual Scoring rules Ver. 2.1 (29) indicated that sleep stage scoring based on single channel EEGs using the same device as that in the present study was comparable to the scoring based on polysomnography according to the AASM Manual Scoring rules Ver. 2.1 (28). Furthermore, sleep stage scoring based on single-channel EEG recording from the frontal pole without EOG and EMG recording was comparable to that with conventional polysomnography, although the EEG device different from, but very similar to, the present device was used (30), which has been previously applied to measurements of REM latency (31). Other sleep components including the time in bed (TIB) (time elapsed from going to bed to final arising), sleep period time (SPT) (time elapsed from sleep onset to the last epoch of sleep), total sleep time (TST) (duration of time spent in NREM and REM during SPT), sleep latency (time elapsed from going to bed to sleep onset), wake time after sleep onset (WASO) (total time of wake stage >60 s after sleep onset), and sleep efficiency (ratio of TST/SPT) were calculated (27). REM sleep onset latency (REM latency) was defined as the time interval between sleep onset and the first occurrence of an epoch of REM sleep. The above parameters to assess sleep architectures were derived from the whole night recording. Based on the EEG sleep stage analysis, a total of 250 min of the data of EEGs, ECGs, and body temperature around sleep onset (5 min before sleep onset and 245 min after sleep onset) were further analyzed in the present study (see below).

Spectral analysis of the EEGs using a fast Fourier transform with a Hanning window was performed every 30 s using commercial software (SleepSign-Lite; KISSEI COMTEC Co., Ltd., Matsumoto, Japan) in the following bands: ultra-slow (0.3–0.8 Hz), delta (0.8–4.0 Hz), theta (4.0–8.0 Hz), alpha (8.0–12.0 Hz), sigma (12.0–16.0 Hz), and beta (16.0–30 Hz). In the individual bands, the mean spectral power density and standard deviation (SD) were computed. The spectral power data in all bands in the epoch in which the mean power of the delta wave exceeded the mean+3SD were excluded from the analysis as noise (32). The 30 s epochs that were assigned as the awake stage were also excluded from the analysis. The mean power was then computed in every 5 min epoch in each band in each subject. The power spectrum data in the 5 min epochs were natural-log transformed for further analyses. The data of the 5 min epochs in each band were also normalized by mean power density across the total sleep period (33).

A total of 250 min of data of the ECGs around sleep onset (5 min before sleep onset and 245 min after sleep onset, i.e., 50 5 min epochs) were analyzed. HRV based on the R-R intervals was analyzed using commercial software with the maximum-entropy method (MemCalc/Win ver2.0; GMS Co., Ltd., Tokyo) (34). Frequency domain spectral analysis of the HRV, which was downsampled to generate a signal to be analyzed for the spectral analysis, was performed on each 120 s epoch, which was shifted by 30 s; the power spectrum was computed every 30 s in a 120 s window (33). The following four power spectrum components were analyzed; very low frequency (VLF) (0.003–0.04 Hz), low frequency (LF) (0.04–0.15 Hz), high frequency (HF) (0.15–0.4 Hz), and total power (0.003–0.4 Hz). Furthermore, the ratio of LF to HF power (LF/HF) was estimated. The mean power spectrum or LF/HF ratio was then computed for every 5 min epoch. These data were natural-log transformed.

The skin temperature of each body region was averaged for every 5 min epoch. The distal-proximal gradient of the skin temperature was calculated by subtracting the proximal temperature (mean temperature between the bilateral subclavicular regions) from the peripheral temperature (mean temperature among the bilateral feet and hands). The difference in distal-proximal gradient of the skin temperature (dDPG) was then computed by subtracting the mean baseline data of distal-proximal gradient of the skin temperature for 30 min before sleep onset from the data after sleep onset.

### Grouping of the Subjects and Statistical Analysis

The subjects were divided into two groups based on subjective fatigue (as determined by the VAS) at wake time on day 3; the low fatigue group (*n* = 12, fatigue VAS score of ≤5.0) and the high fatigue group (*n* = 7, fatigue VAS score of >5.0). The physiological data over 250 min (50 5 min epochs) were analyzed by a two-way analysis of variance (ANOVA) with “group” and “time” as factors. Furthermore, correlations between possible two factors were analyzed by simple linear regression analysis. The significance level was set at *p* < 0.05 except for *post-hoc* multiple comparisons. *Post-hoc* multiple comparisons after two-way ANOVA were performed using Bonferroni test with a significant adjusted *p* < 0.05. The all data were presented as mean values ± standard error of the mean (SEM).

Based on the above correlation analyses, seven autonomic and EEG-related variables were identified as potential factors that might affect the VAS score at wake time (see Results). Furthermore, based on the temporal relationships among these eight variables, a path diagram for the hypothesized set of relationships was created and analyzed by structural equation modeling. Structural equation modeling is a collection of statistical techniques that allow analysis of a set of relationships among multiple factors or measured variables (35). The structural equation modeling analyses were conducted using IBM SPSS AMOS V. 20. To assess the fitness of the hypothetical model, the root mean square error of approximation (RMSEA); comparative fit index (CFI), which is identical to relative non-centrality index; and Tucker-Lewis index (TLI), in addition to χ^2^ values and χ^2^/*df*, were used (36). Furthermore, bootstrap resampling of the original sample was performed using the same software, and the resampled data were re-analyzed by structural equation modeling. The sample size of the resampled data was set at *n* = 10,000 (37).

## Results

### Baseline and Psychological Characteristics

**Figure 2:**
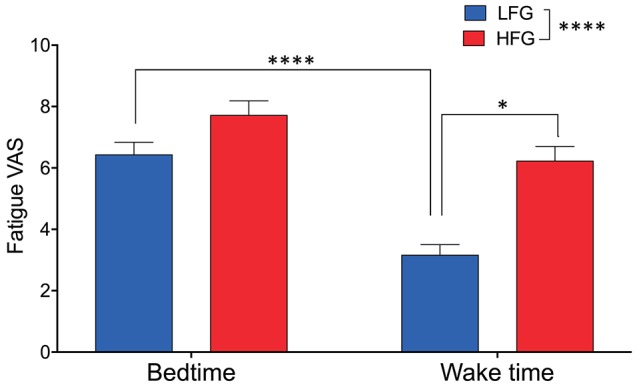
Comparison of subjective feeling of fatigue between the low fatigue and high fatigue groups. LFG, low fatigue group; HFG, high fatigue group. ^*^p < 0.05, ^****^p < 0.0001.

The baseline and psychological data of the subjects are presented in Table 1. There were no significant differences in the baseline characteristics (age, height, body weight, and body mass index, and blood pressure) between the low and high fatigue groups (*t*-test, *p* > 0.05). Furthermore, there were no significant differences in the PSQI, MEQ, and STAI-S scores as well as KSS-J scores at bedtime and wake time between the two groups (*t*-test, *p* > 0.05). In addition, there were no significant differences in sleepiness (responses to SMH questionnaires) between the two groups (*t*-test, *p* > 0.05) (Supplementary Table 1). The high fatigue group reported higher VAS scores (Fatigue VAS) at wake time than did the low fatigue group (*t*-test, *p* < 0.0001) based on definition of the two groups. However, there were no significant differences in the VAS scores at bedtime between the two groups (*t*-test, *p* > 0.05). Furthermore, statistical analysis of the VAS score at bedtime and wake time by a two-way ANOVA with “group” (low vs. high fatigue groups) and “time” (bedtime vs. wake time) as factors indicated that there were significant main effects of group [*F*_(1, 34)_ = 26.95, *p* < 0.0001] and time [*F*_(1, 34)_ = 32.24, *p* < 0.0001], and a significant interaction between group and time [*F*_(1, 34)_ = 4.517, *p* = 0.0409] (Figure 2). *Post-hoc* tests indicated that the VAS score in the low fatigue group was significantly smaller at wake time than at bedtime (Bonferroni test, *p* < 0.0001), and that the VAS score at wake time was significantly smaller in the low fatigue group than in the high fatigue group (Bonferroni test, *p* < 0.05). However, there were no significant differences in the VAS score between bedtime and wake time in the high fatigue group (Bonferroni test, *p* > 0.05). These findings indicated that VAS fatigue was decreased at wake time after sleep in the low fatigue group, but not in the high fatigue group.

**Table 1:** Comparison of the baseline characteristics between the LFG and HFG.

	**LFG (*n* = 12)**	**HFG (*n* = 7)**
Age	32.4 ± 2.7	38.7 ± 3.2
Height (cm)	157.2 ± 1.7	158.9 ± 1.5
Weight (kg)	49.7 ± 1.5	50.9 ± 1.1
BMI	20.1 ± 0.5	20.2 ± 0.6
SBP (mmHg) at bedtime	108.4 ± 4.2	111.9 ± 3.2
DBP (mmHg) at bedtime	69.4 ± 3.0	73.4 ± 2.9
SBP (mmHg) at wake time	111.7 ± 3.5	112.9 ± 4.5
DBP (mmHg) at wake time	68.9 ± 3.3	78.6 ± 4.2
PSQI	5.7 ± 0.6	5.1 ± 0.6
MEQ	54.8 ± 2.4	57.6 ± 3.7
STAI-S at bedtime	42.8 ± 1.7	46.6 ± 1.9
KSS-J at bedtime	5.6 ± 0.5	6.6 ± 0.5
KSS-J at wake time	4.9 ± 0.5	6.3 ± 0.7
Fatigue VAS at bedtime	6.44 ± 0.40	7.72 ± 0.46
Fatigue VAS at wake time	3.17 ± 0.33	6.24 ± 0.46^****^

LFG, low fatigue group; HFG, high fatigue group; SBP, systolic blood pressure; DBP, diastolic blood pressure. Data are presented as mean values ± standard error of the mean (SEM).

**** *Significant difference from LFG (p < 0.0001)*.

### Sleep Architectures and EEG Analysis

Table 2 presents the sleep architectures in the low and high fatigue groups. There were no significant differences in the TIB, SPT, TST, sleep efficiency, WASO, REM duration, and light NREM duration between the low and high fatigue groups (*t*-test, *p* > 0.05). However, the stageN3 sleep duration in the whole sleep period tended to be shorter in the high fatigue group than the low fatigue group (*t*-test, *p* = 0.0505), and the stageN3 sleep duration in sleep cycle 1 was significantly shorter in the high fatigue group than the low fatigue group (*t*-test, *p* < 0.05). Furthermore, the REM latency was significantly shorter in the high fatigue group than in the low fatigue group (*t*-test, *p* < 0.05).

**Table 2:** Comparison of sleep architectures between the LFG and HFG.

	**LFG**	**HFG**
Time in bed (min)	379.7 ± 11.6	367.9 ± 19.7
Sleep period time (min)	351.3 ± 10.3	349.9 ± 17.9
Total sleep time (min)	344.8 ± 9.9	332.3 ± 20.2
Sleep efficiency (%)	98.2 ± 0.2	94.9 ± 2.6
Sleep latency (min)	13.4 ± 2.7	11.1 ± 2.3
**STAGE TIME (MIN)**		
WASO	3.5 ± 0.8	5.2 ± 1.2
REM	78.5 ± 5.6	76.7 ± 9.1
StageN1-2	181.4 ± 8.0	187.3 ± 16.7
StageN3	84.9 ± 4.6	68.3 ± 6.7^#^
StageN3 in sleep cycle 1	36.2 ± 2.0	25.5 ± 3.4^*^
REM latency (min)	72.6 ± 6.5	47.8 ± 7.5^*^

LFG, low fatigue group; HFG, high fatigue group. Data are presented as mean values ± standard error of the mean (SEM).

# Marginal difference from LFG (p = 0.0505);

* *Significant difference from LFG (p < 0.05)*.

**Figure 3:**
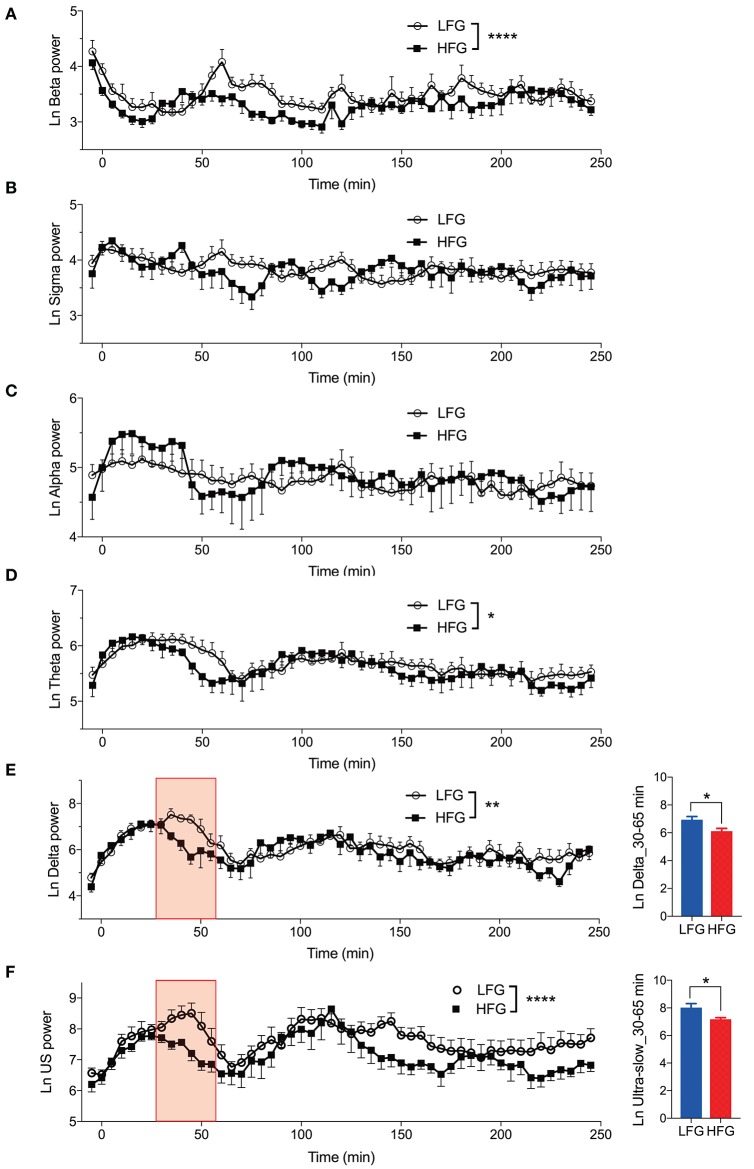
Changes in the electroencephalography powers during sleep. **(A)** Changes in the natural-log transformed beta power (Ln Beta). **(B)** Changes in the natural-log transformed sigma power (Ln Sigma). **(C)** Changes in the natural-log transformed alpha power (Ln Alpha). **(D)** Changes in the natural-log transformed theta power (Ln Theta). **(E)** Changes in the natural-log transformed delta power (Ln Delta). The inset indicates the difference in the mean natural-log transformed delta power from 30 to 65 min (red-colored shaded area) after sleep onset (Ln Delta_30–65 min) between the high fatigue group (HFG) and the low fatigue group (LFG). **(F)** Changes in the natural-log transformed ultra-slow power (Ln US). The inset indicates the difference in the mean natural-log transformed ultra-slow power from 30 to 65 min (red-colored shaded area) after sleep onset (Ln US_30–65 min) between the HFG and LFG. ^*^p < 0.05, ^**^p < 0.01, ^****^p < 0.0001, respectively.

Figure 3 provides the results derived from the power spectrum analysis in each frequency band for the first 250 min of sleep. The data in each frequency band were natural-log transformed; beta power (Ln Beta) (Figure 3A), sigma power (Ln Sigma) (Figure 3B), alpha power (Ln Alpha) (Figure 3C), theta power (Ln Theta) (Figure 3D), delta power (Ln Delta) (Figure 3E), and ultra-slow power (Ln US) (Figure 3F). The data in each frequency band were analyzed by two-way ANOVA with “group” and “time” as factors, and summary of the statistical results was shown in Table 3. The results indicated that there were significant main effects of group as well as time in Ln Beta, Ln Theta, Ln Delta, and Ln US (Table 3). However, there was no significant interaction between group and time in the all frequency bands (Table 3). Furthermore, consistent with the decreased stageN3 duration in sleep cycle 1 in the high fatigue group (Table 2), the mean Ln Delta from 30 to 65 min after sleep onset (Ln Delta_30–65 min) was significantly smaller in the high fatigue group than in the low fatigue group (*t*-test, *p* = 0.0268) (Figure 3E, inset). In addition, the mean Ln US from 30 to 65 min after sleep onset (Ln US_30–65 min) was significantly smaller in the high fatigue group than in the low fatigue group (*t*-test, *p* = 0.0425) (Figure 3F, inset).

**Table 3:** Summary of the statistical results in the EEG data analyses by two-way ANOVA with “group” and “time” as factors in each frequency band.

**Variable**	**Group**		**Time**		**Group** **×** **Time**	
	***F*_**1, 867**_**	***P***	***F*_**50, 867**_**	***P***	***F*_**50, 867**_**	***P***
Ln Beta	30.05	*p* < 0.0001	2.367	*p* < 0.0001	0.7825	0.8616
Ln Sigma	2.041	0.1534	1.131	0.2518	0.9275	0.618
Ln Alpha	1.786	0.1818	1.175	0.1936	0.4448	0.9997
Ln Theta	4.541	0.00334	3.695	*p* < 0.0001	0.5568	0.9946
Ln Delta	9.225	0.0025	5.174	*p* < 0.0001	0.9047	0.6621
Ln US	48.91	*p* < 0.0001	3.505	*p* < 0.0001	0.6254	0.9807

**Figure 4:**
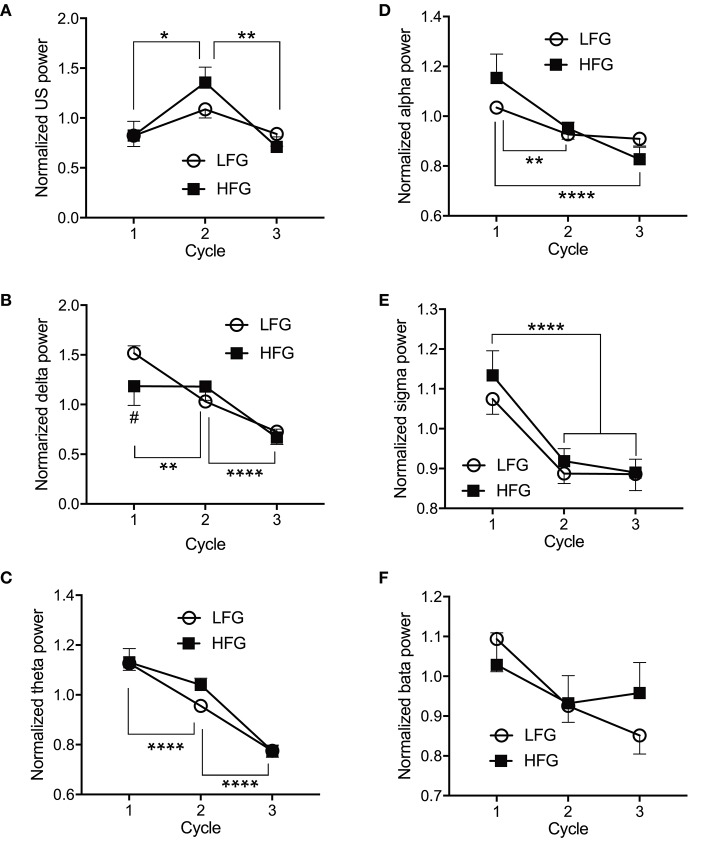
Comparison of the normalized electroencephalography powers across the three initial sleep cycles. **(A)** The normalized ultra-slow (US) power. **(B)** The normalized delta power. **(C)** The normalized theta power. **(D)** The normalized alpha power. **(E)** The normalized sigma power. **(F)** The normalized beta power. LFG, low fatigue group; HFG, high fatigue group; cycle, sleep cycle. ^*^p < 0.05, ^**^p < 0.01, ^****^p < 0.0001, respectively. ^#^Significant difference from the LFG (*p* < 0.05).

Figure 4 presents the normalized EEG powers, relative to the mean power across the whole sleep, in each frequency band across the three sleep cycles. The normalized data in each frequency band were analyzed by two-way ANOVA with “group” and “sleep cycle” as factors, and summary of the statistical results was shown in Table 4. The results indicated that there were significant main effects of sleep cycle in the normalized US, delta, theta, alpha, and sigma powers (Table 4). *Post-hoc* tests for the normalized ultra-slow (US) power indicated that the mean normalized US power was larger in sleep cycle 2 than in sleep cycles 1 and 3 (Bonferroni test, *p* = 0.0104 and 0.0049, respectively) (Figure 4A). *Post-hoc* tests for the normalized delta power indicated that the mean normalized delta power was large r in sleep cycle 1 than in sleep cycle 2, and larger in sleep cycle 2 than in sleep cycle 3 (Bonferroni test, *p* = 0.0020 and *p* < 0.0001, respectively) (Figure 4B). *Post-hoc* tests for the normalized theta power indicated that the mean normalized theta power was larger in sleep cycle 1 than in sleep cycle 2, and larger in sleep cycle 2 than in sleep cycle 3 (Bonferroni test, *p* < 0.0001) (Figure 4C). *Post-hoc* tests for the normalized alpha power indicated that the mean normalized alpha power was larger in sleep cycle 1 than in sleep cycles 2 and 3 (Bonferroni test, *p* = 0.0019 and *p* < 0.0001, respectively) (Figure 4D). *Post-hoc* tests for the normalized sigma power indicated that the mean normalized sigma power was larger in sleep cycle 1 than in sleep cycles 2 and 3 (Bonferroni test, *p* < 0.0001) (Figure 4E). Furthermore, there was a significant interaction between group and sleep cycle only in the normalized delta power (Table 4). The *post-hoc* test for the interaction indicated that the mean normalized delta power was significantly smaller in the high fatigue group than the low fatigue group in sleep cycle 1 (Bonferroni test, *p* = 0.0312).

**Table 4:** Summary of the statistical results in the normalized EEG data analyses by two-way ANOVA with “group” and “sleep cycle” as factors in each frequency band.

**Variable**	**Group**		**Sleep cycle**		**Group** **×** **Time**	
	***F*_**1, 51**_**	***P***	***F*_**2, 51**_**	***P***	***F*_**2, 51**_**	***P***
Normalized beta	0.0829	0.7746	3.004	0.0584	0.7885	0.4600
Normalized sigma	0.8852	0.3512	17.400	*p* < 0.0001	0.2265	0.7981
Normalized alpha	0.3687	0.5464	16.17	*p* < 0.0001	3.052	0.0560
Normalized theta	1.421	0.2387	70.65	*p* < 0.0001	1.245	0.2966
Normalized delta	1.254	0.2697	27.89	*p* < 0.0001	3.754	0.0301
Normalized US	0.2294	0.6340	7.974	0.0010	1.367	0.2641

**Figure 5:**
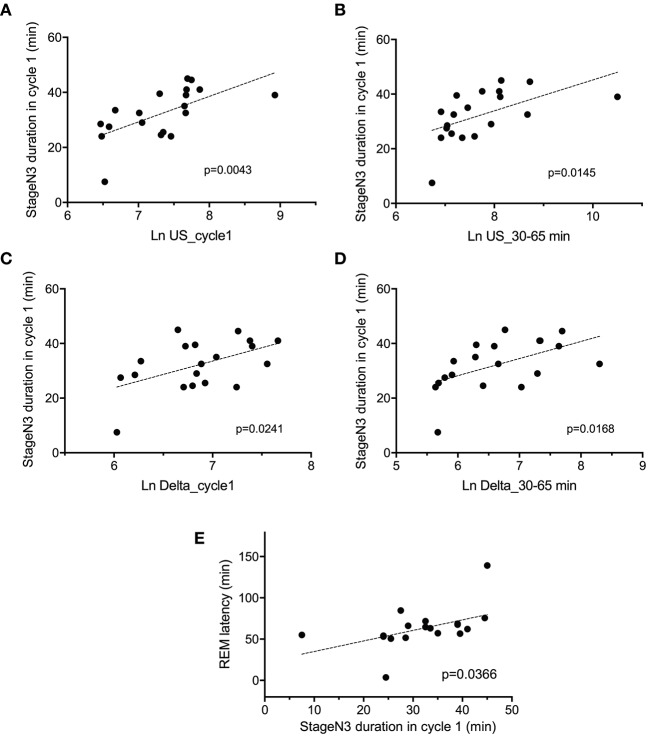
Correlation between the deep non-rapid eye movement sleep duration in sleep cycle 1 and the electroencephalography parameters. Correlations of the stageN3 duration in sleep cycle 1 (stageN3 duration in cycle 1) with **(A)** the mean natural-log transformed ultra-slow powers in sleep cycle 1 (Ln US_cycle1), **(B)** the mean natural-log transformed ultra-slow powers from 30 to 65 min after sleep onset (Ln US_30–65 min), **(C)** the mean natural-log transformed delta powers in sleep cycle 1 (Ln Delta_cycle1), **(D)** the mean natural-log transformed delta powers from 30 to 65 min after sleep onset (Ln Delta_30–65 min), and **(E)** the REM latency.

The above results indicated that there were significant differences in the four parameters [the stageN3 sleep duration in sleep cycle 1, REM latency, and slow waves (delta and ultra-slow waves)] between the two groups. Figure 5 indicates the relationships between these parameters. Statistical analyses by simple regression analysis indicated that the stageN3 sleep duration in sleep cycle 1 (stageN3 duration in cycle 1) was significantly and positively correlated with the mean Ln US in sleep cycle 1 (Ln US_cycle1) [*F*_(1, 17)_ = 10.86, *p* = 0.0043] (Figure 5A) and those from 30 to 65 min after sleep onset (Ln US_30–65 min) [*F*_(1, 17)_ = 7.412, *p* = 0.0145] (Figure 5B). Furthermore, statistical analyses by simple regression analysis indicated that the stageN3 duration in cycle 1 was significantly and positively correlated with the mean Ln Delta in sleep cycle 1 (Ln Delta_cycle1) [*F*_(1, 17)_ = 6.128, *p* = 0.0241] (Figure 5C) and the Ln Delta_30–65 min [*F*_(1, 17)_ = 7.027, *p* = 0.0168] (Figure 5D). In addition, statistical analysis by simple regression analysis indicated that the REM latency was significantly and positively correlated with the stageN3 duration in cycle 1 [*F*_(1, 17)_ = 5.145, *p* = 0.0366] (Figure 5E). These results suggest that the decrease in the stageN3 duration in sleep cycle 1 and the REM latency in the high fatigue group (Table 2) might be ascribed to the decrease in the ultra-slow and delta powers in the high fatigue group.

### Autonomic Activity During Sleep

**Figure 6:**
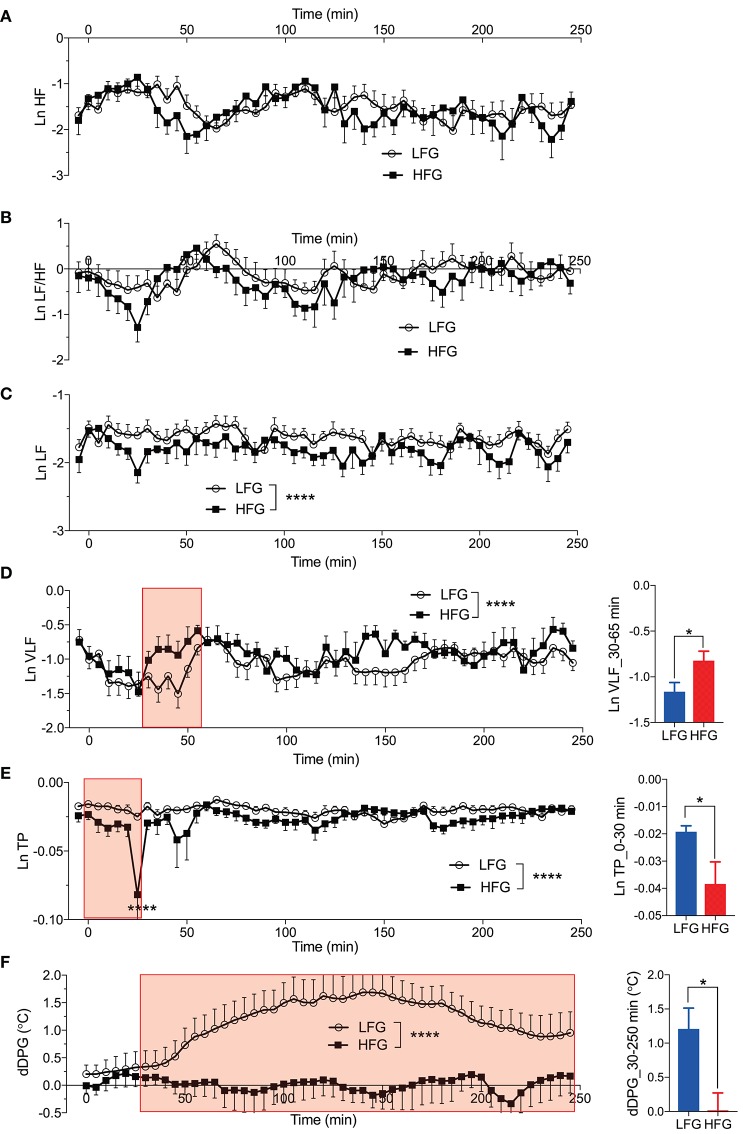
Changes in the autonomic parameters during sleep. **(A)** Changes in the natural-log transformed high frequency (HF) (Ln HF). **(B)** Changes in the natural-log transformed low frequency (LF)/HF (Ln LF/HF). **(C)** Changes in the natural-log transformed LF (Ln LF). **(D)** Changes in the natural-log transformed very low frequency (VLF) (Ln VLF). The inset indicates the comparison of the mean Ln VLF from 30 to 65 min (red-colored shaded area) after sleep onset (Ln VLF_30–65 min) between the low fatigue group (LFG) and the high fatigue group (HFG). **(E)** Changes in the natural-log transformed total power (Ln TP). The inset indicates the comparison of the mean Ln TP from 0 to 30 min (red-colored shaded area) after sleep onset (Ln TP_0–30 min) between the HFG and the LFG. **(F)** Changes in the difference distal-proximal gradient (dDPG). The inset indicates the comparison of the mean dDPG from 30 to 250 min (red-colored shaded area) after sleep onset (dDPG_30–250 min) between the HFG and LFG. ^*^p < 0.05, ^****^P < 0.0001, respectively.

Figures 6A–E presents the changes in natural-log transformed HRV parameters during sleep in the low and high fatigue groups. The natural-log transformed HRV data were analyzed by two-way ANOVA with “group” and “time” as factors, and summary of the statistical results was shown in Table 5. The statistical results indicated that there were significant main effects of group in the natural-log transformed LF (Ln LF), natural-log transformed VLF (Ln VLF), and natural-log transformed total power (Ln TP) (Table 5), while there were significant main effects of time in the natural-log transformed HF (Ln HF), Ln VLF, and Ln TP (Table 5). Furthermore, there was a significant interaction between group and time only in Ln TP (Table 5). *Post-hoc* tests for the interaction indicated that the Ln TP was smaller in the high fatigue group than in the low fatigue group in the 7th epoch (from 25 to 30 min after sleep onset) (Bonferroni test, *p* < 0.0001). In addition, the mean Ln VLF from 30 to 65 min after sleep onset (Ln VLF_30–65 min) was significantly greater in the high fatigue group than in the low fatigue group (*t*-test, *p* = 0.0400) (Figure 6D, inset), while the mean Ln TP from 0 to 30 min after sleep onset (Ln TP_0–30 min) was significantly smaller in the high fatigue group than in the low fatigue group (*t*-test, *p* = 0.0108) (Figure 6E, inset).

**Table 5:** Summary of the statistical results in the autonomic data analyses by two-way ANOVA with “group” and “time” as factors in each frequency band.

**Variable**	**Group**		**Time**		**Group** **×** **Time**	
	***F*_**1, 867**_**	***P***	***F*_**50, 867**_**	***P***	***F*_**50, 867**_**	***P***
Ln HF	0.9834	0.3216	1.758	0.0012	0.6499	0.9715
Ln LF/HF	3.522	0.0609	1.228	0.1377	0.5173	0.9978
Ln LF	34.03	*p* < 0.0001	1.036	0.4074	0.4802	0.9992
Ln VLF	17.36	*p* < 0.0001	1.674	0.0028	0.6568	0.9685
Ln TP	36.31	*p* < 0.0001	1.808	0.0007	1.475	0.0195
dDGP	222.5	*p* < 0.0001	0.7557	0.8907	1.055	0.3737

In the dDPG (Figure 6F), statistical analysis by two-way ANOVA indicated that there was a significant main effect of group, but no significant interaction between group and time (Table 5). Furthermore, the mean dDPG from 30 to 250 min after sleep onset (dDPG_30–250 min) was significantly greater in the low fatigue group than in the high fatigue group (*t*-test, *p* = 0.0163) (Figure 6F, inset). These results indicated that there were also significant differences in the autonomic parameters during sleep between the low and high fatigue groups.

### Relationships Between the VAS Score and the EEG and Autonomic Parameters

**Figure 7:**
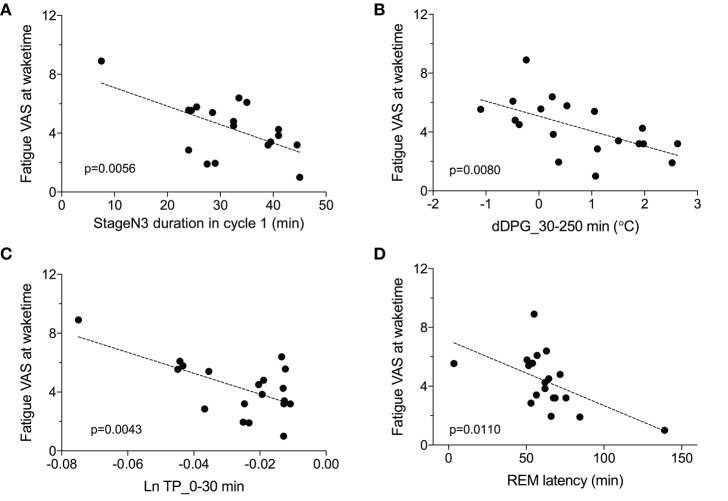
Parameters correlated with fatigue as determined by the visual analog scale at wake time (Fatigue VAS at waketime). **(A)** Correlation with the stageN3 duration in sleep cycle 1 (stageN3 duration in cycle 1). **(B)** Correlation with the mean difference distal-proximal gradient (dDPG) from 30 to 250 min after sleep onset (dDPG_30–250 min). **(C)** Correlation with the mean natural-log transformed total power from 0 to 30 min after sleep onset (Ln TP_0–30 min). **(D)** Correlation with the rapid eye movement (REM) latency.

We then analyzed the relationships between the fatigue VAS score at wake time and the EEG and autonomic parameters with significant differences between the low and high fatigue groups. Statistical analyses by simple regression analyses indicated that four parameters were found to be significantly correlated with fatigue as evaluated by the VAS at wake time (Figure 7); fatigue VAS at wake time was significantly and negatively correlated with the stageN3 duration in cycle 1 [*F*_(1, 17)_ = 10.02, *p* = 0.0056] (Figure 7A) and the dDPG_30–250 min [*F*_(1, 17)_ = 9.006, *p* = 0.0080] (Figure 7B). Furthermore, fatigue VAS at wake time was significantly and negatively correlated with the Ln TP_0-30 min [*F*_(1, 17)_ = 10.83, *p* = 0.0043] (Figure 7C) and the REM latency [*F*_(1, 17)_ = 8.151, *p* = 0.0110] (Figure 7D).

**Figure 8:**
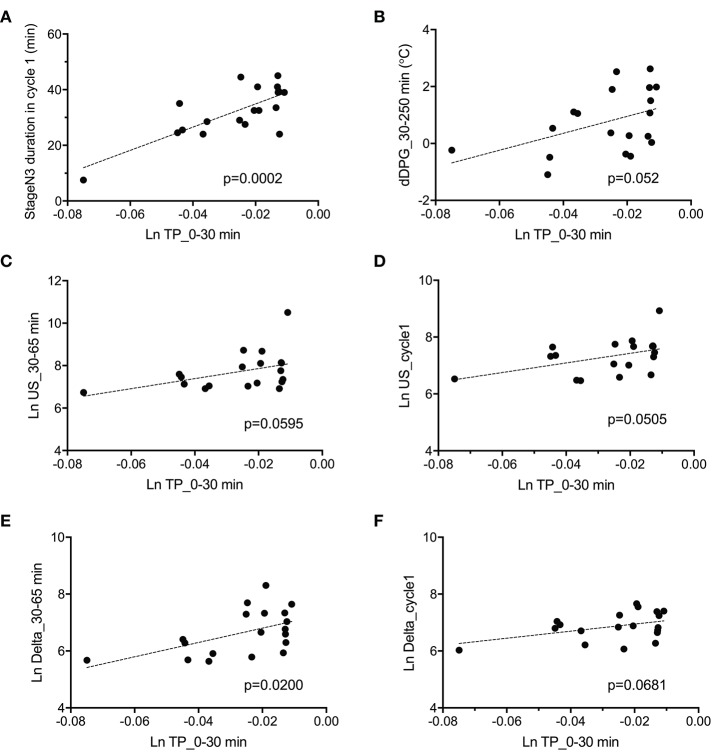
Parameters correlated with total power during the initial phase of sleep (Ln TP_0–30 min). **(A)** Correlation with the stageN3 duration in sleep cycle 1 (stageN3 duration in cycle 1). **(B)** Correlation with the mean difference distal-proximal gradient (dDPG) from 30 to 250 min after sleep onset (dDPG_30–250 min). **(C)** Correlation with the mean natural-log transformed ultra-slow power (Ln US) from 30 to 65 min after sleep onset (Ln US_30–65 min). **(D)** Correlation with the mean Ln US in sleep cycle 1 (Ln US_cycle1). **(E)** Correlation with the mean natural-log transformed delta power (Ln Delta) from 30 to 65 min after sleep onset (Ln Delta_30–65 min). **(F)** Correlation with the mean Ln Delta in sleep cycle 1 (Ln Delta_cycle1).

The above data in Figure 7 indicate that fatigue VAS at wake time was correlated with four parameters (stageN3 duration in cycle 1, dDPG_30–250 min, Ln TP_0–30 min, and REM latency). Furthermore, stageN3 duration in cycle 1 was correlated to Ln US_cycle1, Ln US_30–65 min, Ln Delta_cycle1, and Ln Delta_30–65 min (Figure 5). Among these parameters, the Ln TP_0–30 min underwent the earliest changes. This suggests that changes in the remaining parameters might be induced by changes in Ln TP_0–30 min. We analyzed this possibility; Figure 8 demonstrates the relationships between the Ln TP_0–30 min and other factors. Statistical analyses by simple regression analysis indicated that the Ln TP_0–30 min was significantly and positively correlated with the stageN3 duration in cycle 1 [*F*_(1, 17)_ = 22.67, *p* = 0.0002] (Figure 8A), and tended to be positively correlated with the dDPG_30–250 min [*F*_(1, 17)_ = 4.364, *p* = 0.052] (Figure 8B). The significant correlation of the Ln TP_0–30 min with the stageN3 duration in cycle 1 might be mediated via the effect of the Ln TP_0–30 min on the ultra-slow and delta powers. Consistent with this notion, the Ln TP_0–30 min tended to be positively correlated with the Ln US_30–65 min [*F*_(1, 17)_ = 4.079, *p* = 0.0595] (Figure 8C), and with the mean Ln US_cycle1 [*F*_(1, 17)_ = 4.431, *p* = 0.0505] (Figure 8D). Furthermore, the Ln TP_0–30 min was significantly and positively correlated with the Ln Delta_30–65 min [*F*_(1, 17)_ = 6.585, *p* = 0.0200] (Figure 8E), and tended to be positively correlated with the Ln Delta_cycle1 [*F*_(1, 17)_ = 3.795, *p* = 0.0681] (Figure 8F).

**Figure 9:**
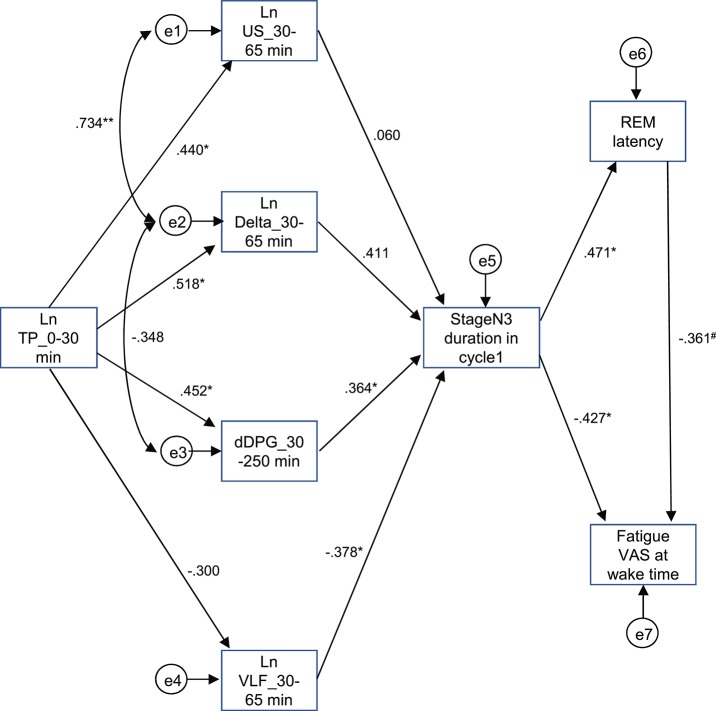
Diagram and results derived from structural equation modeling with eight observed variables. Single-headed arrows (paths) indicate the causal relationships in the model, with the variable at the tail of the arrow causing the variable at the point. Double-headed arrows indicate covariances or correlations, without a causal interpretation. Statistically, the single-headed arrows (paths) represent regression coefficients, while the double-headed arrows represent covariances. e1-8, measurement errors. Values near the arrows indicate standardized regression coefficients. ^*^p < 0.05, ^**^p < 0.01, ^#^*p* < 0.1.

**Figure 10:**
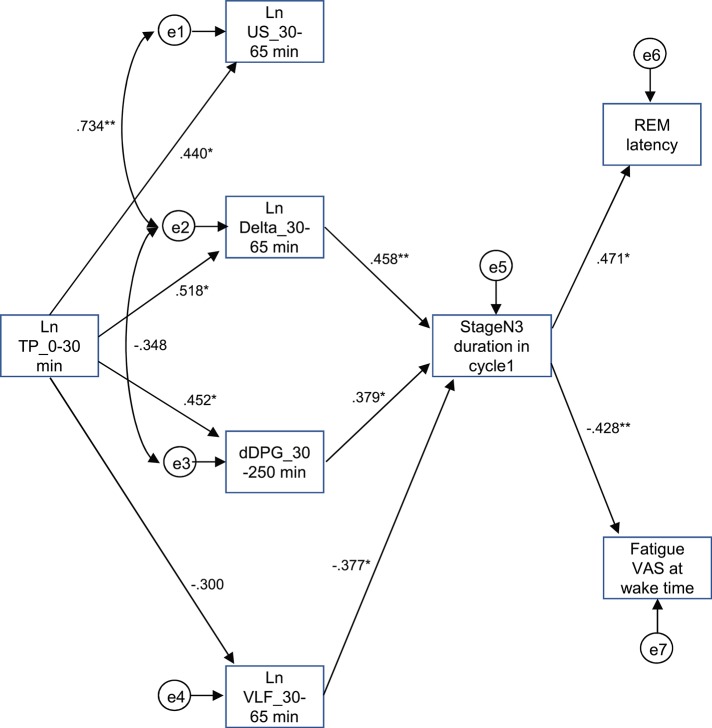
Modified diagram and results derived from structural equation modeling with eight observed variables. Description is the same as that for Figure 9. ^*^p < 0.05, ^**^p < 0.01.

The above results identified the six possible variables (the Ln TP_0–30 min, Ln US_30–65 min, Ln Delta_30–65 min, dDPG_30–250 min, stageN3 duration in cycle 1, and REM latency) with an additional factor of LnVLF_30–65 min that was increased in the high fatigue group, which might affect fatigue VAS at wake time. Based on the time when the variables changed and the linear relationships between the variables, we hypothesized that the initial decrease in the Ln TP_0–30 min might increase fatigue VAS at wake time via the changes in the Ln US_30–65 min, Ln Delta_30–65 min, dDPG_30–250 min, Ln VLF_30–65 min, stageN3 duration in cycle 1, and REM latency (Figure 9) (see the Discussion). This hypothetical model was analyzed by structural equation modeling. The overall fit indices of the model indicated that fitting with the data was acceptable [χ^2^ = 14.623, (*p* = 0.479); *df* = 15; χ^2^/*df* = 0.975; CFI = 1.000; RMSEA = 0.000; TLI = 1.013]. However, it was noted that the path from the Ln US_30–65 min to the stageN3 duration in cycle1 and another path from the REM latency to fatigue VAS at wake time was not significant (*p* > 0.05). We then analyzed the modified model in which these two paths were deleted (Figure 10). The results indicated that fitting with the data was acceptable [χ^2^ = 17.756, (*p* = 0.404); *df* = 17; χ^2^/*df* = 1.044; CFI = 0.986; RMSEA = 0.050; TLI = 0.977]. All paths aside from the Ln TP_0–30 min to the Ln VLF_30–65 min path were significant (*p* < 0.05). We further analyzed this modified model by bootstrapping (*n* = 10,000). The bootstrapping data confirmed the same results as those in the original data (*n* = 19); all paths aside from the Ln TP_0–30 min to the Ln VLF_30–65 min path were significant.

## Discussion

### Physiological Differences Between the Low and High Fatigue Groups

In the present study, the fatigue VAS score was lower at wake time in the low fatigue group, but not in the high fatigue group, suggesting that the low fatigue group recovered from fatigue during sleep, but the high fatigue group did not recover; i.e., “non-restorative sleep” occurred in the high fatigue group. The high fatigue group exhibited the following changes compared to the low fatigue group; (1) a decrease in the stageN3 sleep duration in sleep cycle 1; (2) a decrease in the ultra-slow, delta, theta, and beta powers; (3) a decrease in the REM latency; (4) a decrease in the LF and total power of HRV; (5) an increase in the VLF; and (6) a smaller increase in the dDPG. Consistent with the present results, previous studies reported that a decrease in the ultra-slow and delta powers occurs in chronic fatigue syndrome (CFS) (6, 38). These findings suggest that the changes in the EEG powers in the high fatigue group might affect recovery from fatigue during sleep.

In addition to the decreased mean REM latency in the high fatigue group, the one subject showed very short REM latency (<8 min) in the high fatigue group (Figure 7D), and could be narcoleptic. However, it is unlikely since the decreased REM latency in the high fatigue group could be ascribed to sleep loss (Total sleep time <6 h) and/or shift work in the present study. Previous studies reported that both sleep loss and shift work were significantly associated with short onset of REM sleep (<8–15 min) in a nocturnal polysomnography and Sleep-Onset REM (SOREM) in a daytime multiple sleep latency test (MSLT) (39, 40), which can lead to a false positive diagnosis for narcolepsy (39, 40). Thus, the present results are consistent with these previous studies, and the given subject with short REM latency <8 min in the present study might be non-narcoleptic since majority of subjects with positive findings in the MSLT seemed to be false positive in diagnosis of narcolepsy especially in female subjects (39). However, it is noted that the sleep stage scoring in the present study was based on single-channel EEGs, and the present results should be interpreted with caution (see Conclusions and limitations).

Interestingly, Ln TP and Ln LF, which were significantly decreased in the high fatigue group, were relatively stable over 250 min. In contrast, Ln HF showed cyclic changes similar to Ln Delta that correlates with sleep depth or sleep stage by definition, while Ln LF/HF and Ln VLF showed opposite patters to that of Ln Delta. These findings are consistent with previous studies in which sympathetic-dominant activity (e.g., Ln LF/HF, Ln VLF) was negatively correlated with sleep stages, while vagal-dominant activity (e.g., Ln HF) was positively correlated with sleep stages (41, 42). Since LF and total power of HRV include both parasympathetic and sympathetic activity (43–45), these two variables could be less prone to show sleep stage-related changes. Second, some studies reported changes in total power of HRV, which was negatively correlated with urine levels of cortisol and noradrenaline and saliva cortisol levels (46), and a decrease in the LF and total power of HRV in athletes with fatigue and patients with CFS (4, 47), suggesting that total power of HRV is reduced in stressed or exhausted conditions.

Furthermore, the distal-proximal gradient of the skin temperature is an indirect measure of heat dissipation or heat loss from the core (brain) to the periphery (hand and leg skin) due to vasodilation in the peripheral skin, which is associated with sleepiness and the body's readiness for sleep as well as NREM 48–50. The smaller increase in the dDPG in the high fatigue group suggests that heat dissipation to lower the core temperature leading to NREM sleep was less evident in the high fatigue group. Furthermore, the VLF was increased in the high fatigue group. It is reported that an increase in the VLF reflects thermogenesis by an increase in metabolism (51, 52), suggesting that thermogenesis was increased in the high fatigue group. These two physiological processes (an increase in thermogenesis and lowered heat dissipation) might increase the core temperature, which might in turn have decreased NREM sleep in the high fatigue group.

### Physiological Mechanisms of Fatigue at Wake Time

The difference in the EEG and autonomic parameters between the low and high fatigue groups may be due to the individual differences in physiological reactions to shift-work conditions. The above inference suggests that the non-recovery from fatigue due to “non-restorative sleep” in the high fatigue group might be ascribed to the changes in the EEG and HRV parameters. Consistent with this notion, fatigue as determined by the VAS at wake time was negatively correlated with (1) the mean Ln TP_0–30 min), (2) stageN3 duration in cycle 1, (3) mean dDPG_30–250 min, and (4) REM latency (Figure 7). Among these four factors correlated with fatigue as determined by VAS, Ln TP_0–30 min underwent the earliest changes, and was correlated with the stageN3 duration in cycle 1 and dDPG_30–250 min (Figures 8A,B). Furthermore, (1) the Ln TP_0–30 min was correlated with the EEG parameters in NREM sleep in sleep cycle 1 (Ln US_30–65 min, Ln US_cycle1, Ln Delta_30–65 min, Ln Delta_cycle1) (Figures 8C–F), and (2) the stageN3 duration in cycle 1 was correlated with the REM latency (Figure 5E). These findings suggest that the Ln TP_0–30 min might affect fatigue VAS at wake time indirectly, via the remaining five factors (the dDPG_30–250 min, Ln US_30–65 min, Ln Delta_30–65 min, stageN3 duration in cycle 1, and REM latency). In addition, the Ln VLF_30–65 min, which was also increased in the high fatigue group, might also mediate effects of the Ln TP_0–30 min. The structural equation modeling results support this initial hypothesized model (Figure 9). However, the modified model indicated that the Ln US_30–65 min and REM latency might not be directly related to fatigue VAS at wake time (Figure 10).

There is some evidence that pro-inflammatory cytokines (e.g., IL-1β, TNF-α, IL-6) reduce HRV including HF, LF, and total power of HRV (53–56), and extensive studies have reported that various cytokines are associated with the pathogenesis of fatigue in various diseases such as CFS, cancers, and multiple sclerosis (57–59). Although the subjects in the present study were healthy adult women without such diseases, it is reported that the serum levels of cytokines increase in response to various stresses including psychological stress (60), and serum cytokine levels are also affected by circadian rhythms and increase as early as 30 min after sleep onset in healthy subjects (61). Furthermore, administration of IL-6 resulted in a decrease in the first half of NREM sleep in humans (62), which was consistent with the decrease in the stageN3 sleep duration in sleep cycle 1 in this study. IL-6 was also found to increase body temperature in humans (62), and IL-6 levels in the cerebrospinal fluid were correlated with an increase in body temperature in rats (63), which was consistent with the reported increase in the VLF associated with thermogenesis (51, 52). In addition, animal experiments suggest that sleep, body temperature, and cardiovascular functions are controlled by distinct groups of neurons within the preoptic area of the hypothalamus, and different information from these groups of neurons are integrated within this nucleus as well as by an interaction with global networks for homeostasis (64, 65). Furthermore, a complex interaction between brain areas controlling autonomic nervous system and those controlling sleep-wake state has been proposed (66). These findings suggest that increased levels of pro-inflammatory cytokines in response to various stressors during the day might induce a decrease in the total power of HRV during the initial phase of sleep, which in turn might induce various changes in the EEG and autonomic parameters as well as fatigue VAS at wake time through activity changes in the preoptic area of the hypothalamus.

## Conclusions and Limitations

The present results indicated that the high fatigue group did not recover from fatigue, which might be mediated through the following sequential three processes. First, the present results along with the previous studies suggest that an increase in peripheral pro-inflammatory cytokines might suppress total power of HRV, which might be mediated through their direct effects on the autonomic control areas such as the hypothalamus and/or indirect effects via peripheral afferent nerves (67, 68). Second, the correlational and structural equation modeling analyses indicated that an initial decrease in total power of HRV decreased the stageN3 sleep duration in sleep cycle 1 via its effects on other autonomic and EEG parameters including delta power and dDPG. It is reported that the autonomic nervous system densely interacts with the hypothalamus including the preoptic area controlling wakefulness-sleep cycle as well as body temperature (64–66). Therefore, the initial decrease in total power of HRV might affect dDPG and stageN3 sleep duration in sleep cycle 1 through the complex interactive pathways between the two systems. Third, the decrease in the stageN3 sleep duration in sleep cycle 1 might inhibit the recovery from fatigue during sleep (see below).

Sleep is important for homeostasis as a restorative process (69–71); cell damages are repaired, brain wastes are cleared, and macromolecules/neurotransmitters are restored during sleep. The present results indicated that the high fatigue group with short stageN3 sleep duration in sleep cycle 1 did not recover from fatigue. These findings suggest that stageN3 sleep in sleep cycle 1 is important for recovery from disturbed physiological conditions in the brain and body due to activity during wakefulness (e.g., cell damages, increases in wastes, shortages of macromolecules/neurotransmitters), while the disturbed physiological conditions did not fully recover, and the physiological conditions remained disturbed until wake time in the high fatigue group with short stageN3 sleep duration in sleep cycle 1. The sleep cycle 1 might be important for the recovery since stageN3 sleep period is usually longest in this sleep cycle. Consistent with this notion, protein synthesis during sleep was positively correlated to ratios of deep NREM (stageN3) sleep in that sleep although differences in protein synthesis among different sleep cycles remain unknown, suggesting that stageN3 sleep is important for restorative sleep (72). Furthermore, previous studies have reported that warming of the periocular and posterior cervical regions increased the delta power in the first 90 min of the sleep episode and decreased fatigue at wake time (73, 74). Further studies are required to investigate the selective role of sleep cycle 1 in recovery from fatigue.

However, the present study has several limitations. The number of enrolled subjects was relatively small. Second, sleep scoring was performed based on a single EEG derivation instead of conventional polysomnography in the present study. Third, since the physiological recordings were performed at home instead of in a temperature-controlled room to avoid psychological stress due to the new environment, differences in the environmental conditions across the subjects might have affected the results. Fourth, since the data in fixed length epochs of 5 min were quantitatively analyzed in the present study, different data during different sleep (REM and NREM) stages could be mixed in the same epochs. Fifth, we recorded only the limited number of the variables; other factors including the serum cytokine levels of the subjects and neural activity in various brain areas controlling sleep-wakefulness and autonomic activity (66, 75) were not measured. Sixth, we used the subjects with relatively short sleep period time (<6 h) in the present study. Nurses in the high fatigue group, who could sleep for longer than 6 h in different shift work situations, could recover from fatigue at bedtime. Seventh, effects of prior shift works were not considered in the present study. Previous studies suggest that balance between NREM and REM sleeps is homeostatically controlled (76, 77), and homeostatic and circadian systems combine to affect awake-sleep cycle (13, 78). Therefore, prior shift works could affect fatigue recovery during sleep through a complex interaction between these two systems. Further studies with a larger number of subjects of different sexes, occupations, and work shift patterns including no shift work as well as those with conventional polysomnography are required in order to generalize the present results of the relationships between fatigue and sleep parameters. Nevertheless, the present results provide clues to the underlying mechanisms and treatment of fatigue after sleep.

## Author Contributions

TI and HisN designed research. SG and HisN performed research. SG, TI, JM, YT, EH, YN, TT, TO, and HisN analyzed data. SG, TI, HirN, and HisN wrote the manuscript.

### Conflict of Interest Statement

The authors declare that the research was conducted in the absence of any commercial or financial relationships that could be construed as a potential conflict of interest.
