# Supplementary material for: Non-restorative Sleep Caused by Autonomic and Electroencephalography Parameter Dysfunction Leads to Subjective Fatigue at Wake Time in Shift Workers
Source: Front Neurol. 2019 Feb 5;10:66. doi: 10.3389/fneur.2019.00066 (PMC6370690; doi:10.3389/fneur.2019.00066)
Supplement: Supplementary file 1 [file Data_Sheet_1.PDF]

Supplementary Material

Non-restorative sleep caused by autonomic and electroencephalography parameter dysfunction leads to subjective fatigue at wake time in shift workers

Sofya Gorlova, Tomohisa Ichiba, Hiroshi Nishimaru, Yusaku Takamura, Jumpei Matsumoto, Etsuro Hori, Yoshinao Nagashima, Tsuyoshi Tatsuse, Taketoshi Ono, Hisao Nishijo*

*** Correspondence:** Dr. Hisao Nishijo, nishijo@med.u-toyama.ac.jp

1 Supplementary Table 1

Supplementary Table 1. Comparison of responses to the SMH questionnaires between the LFG and HFG.

Questionnaire		LFG (n=12)	HFG (n=7)
How was your Sleep?	[light (1) - deep (7)]	4.8 ± 0.3	4.3 ± 0.3
How many times did you wake up?	[time(s)]	2.5 ± 0.3	3.1 ± 0.4
How well did you sleep last night?	[badly (1) - well (6)]	4.0 ± 0.2	3.6 ± 0.4
How clear-headed did you feel after getting up this morning?	[still very drowsy (1) - alert (6)]	3.2 ± 0.3	2.4 ± 0.3
How satisfied were you with last night's sleep?	[unsatisfied (1) - satisfied (5)]	3.5 ± 0.2	2.9 ± 0.3
How much difficulty did you have in getting off to sleep last night?	[none or very little (1) - difficult (4)]	1.6 ± 0.1	1.4 ± 0.2

There were no significant differences in the all questionnaires between the LFG and HFG (t-test, $p > 0.05$).
